# Selections

**Published:** 1860-10

**Authors:** 


### Selections.

*Salivary Calculus.*—J. H., aged 48 years, of spare habit and slender constitution, some fourteen years since was seized with a severe pain under the left side of his tongue. He applied to his family physician, who could give him no satisfactory information as to the cause or nature of his complaint; neither could he afford him any relief. He was induced to consult other physicians in his vicinity, and he did so with like results. In the mean time, a small tumor made its appearance on the under side of his tongue, near or at the seat of pain. He went to Boston and consulted the late Dr. ——, who informed him that his disease was cancer, and gave him but little encouragement as to any permanent relief. He returned to his home, determined to abide the result of what he then supposed to be an incurable disease. From that time until about the first of February last, he has suffered paroxysms of severe and excruciating pain at different times

The tumor gradually increased in size, and the paroxysms of pain became more frequent, until it finally became inflamed, suppurated and burst, discharging a small quantity of pus and a calculus weighing fifteen grains, having the general appearances of ordinary renal or biliary calculi. He has since been entirely free from pain.—*Boston Med. and Surg. Journal.*

---

*The Turkish Bath.*—In these Turkish baths, soap and water are purely secondary agents; they are considered as barbarous, clumsy, and effete means of cleansing. The bather is first conducted into a room, which is practically a large oven, lighted from the top, and filled with moist air. This is very far, however, from being a vapor bath; the quantity of water-vapor is small, and does not affect the transpiration of water by the skin. Of course, a profuse sweating is induced, and the skin is thoroughly softened. It is a hot bath without water, or rather with the aid of very little water. From this chamber he passes to another—the calidarium—where, freely perspiring, he is rubbed with towels or goat's hair gloves; and so great is the effect of the prior treatment, that the softened cuticle rolls off in thick flakes, and a new skin is found beneath, of which the subject of the operation little dreamed. No one who takes a Turkish bath for the first time but must be astonished at the quantity of the unnecessary cuticle which he carries about with him. Adepts tell you that "it requires great dexterity to perform this well without rubbing some places too much, and others too little." Now comes a drenching with warm water and soap, which is not the most agreeable part of the bath, and may be considered partially unnecessary. Then the bather passes back to the tepidarium, where he is dried and clothed in warm towels; and, after a pause, thence to his frigidarium, or cool chamber, where, still clothed in warm towels, he sips coffee, smokes a narghilet, and indulges in beatific sensations which only those can know who have passed through the three purgatories of the bath. The Turkish bath is an agent of such great power in restoring the active functions of the skin, and the ordinary results of its application are so peculiarly agreeable and invigorating, that it will probably invite the attention of medical practitioners in its relations to disease. It is a powerful agent, of which the virtues are apparent; but incautiously employed by persons liable to congestion of the head or organs of the chest, it is not free from dangers, as some unfortunate circumstances have already proved.—*Lancet.*

### DEVELOPMENT OF THE TEETH OF CATTLE, AND MODE OF ASCERTAINING THEIR AGE BY THE SAME.

Persons acquanted with the dentition of "neat stock," can form a pretty accurate idea of age, from the period of birth up to that of adult life; and this method of ascertaining the age of an animal is, probably, more correct than that which applies to *horns*; for by means of a rasp applied to the rings of the horns, any amount of imposition may be practiced, when it is well known that the same liberties are not to be taken with the teeth without the chances of discovery. It is possible that there may be some slight variations from the following rules, in the development of the teeth, yet such variations will not embrace a period of over a month or six weeks, which at maturity does not amount to much, and may be considered as purely accidental—out of the ordinary course of nature. The front teeth, or temporary incisors, are found in the lower jaw; there are eight of them, all prominent at the age of four weeks. The calf is usually born with three temporary grinders or molars; the fourth appears six months after birth; the fifth appears at the age of fifteen months; and the sixth is to be seen at the age of two and a half years; now, the animal has a "full mouth" of temporary teeth, numbering thirty-two. At this period a very remarkable change in the teeth is about to occur; the temporary ones, having answered the purposes for which they were intended, are to be removed in the following order, so as to give place to others which shall correspond to the increase in the size of the jaw bones, and proved as durable as other bones of the body. At the age of two years, the central or middle incisors (lower jaw) are shed and replaced by two permanent ones. At the age of three, the two incisors known as the inner middle, undergo the same process. At the age of four the outer middle are shed, and replaced by permanent teeth. At the age of five, the corner incisors are also transformed in the same manner, and the animal has a full set (eight) of permanent front teeth. The first and second permanent molars, known as grinders, appear in the upper and lower jaws, on each side, at the age of two years; and at intervals of one year, the other four are successively cut; so that at the age of six years, the animal has a "full mouth" of permanent grinders.—*American Stock Journal.*

### RETURN OF SENSIBILITY AFTER SECTION OF NERVES.

BY DR. LOTZBECK, OF TUBINGEN.

The author has devoted himself to minute researches on the return of sensibility in five cases of the division of nerves, three of which belonged to the inferior maxillary, the other to the infraorbital. In four cases a portion of nerve more or less had been exsected, and in one case simple section only was performed.

In all the cases the operation had been followed within the sphere of distribution of the divided nerve, by a diminution of, or total insensibility to touch, and the perception of temperature; this modification of sensibility has been complete, either immediately after the operation, or from twenty-four to forty-eight hours subsequently, or at most only twelve days.

The diminution of perception of temperature is sometimes proportioned to the abolition of tactile sensibility, and makes the same progress; sometimes it continues even when the tactile sensibility has already perceptibly diminished, and does not disappear entirely until a subsequent period.

The diminution of tactile sensibility is accompanied by the following particulars: the patients perceive more slowly and with less precision the part that is touched; besides, in order to produce a double impression, it is necessary that the two points of a compass, placed in contact with the skin, be farther separated than in the normal condition, and that the distance which it is necessary to leave between the two points be gradually increased.

In all the cases observed, a return of sensibility, more or less perceptible, has been verified. This phenomenon has, moreover, exhibited wide variations in the different cases; this has been observed sometimes after some days, sometimes after the lapse of a much longer period; sensibility may be restored in the entire extent of the integument where it had disappeared, or only within circumscribed limits; sometimes it returns to its normal level, sometimes it remains weak.

The return of the perception of temperature proceeds generally, and with almost trifling variations *pari passu*, with the restitution of tactile sensibility; but in one case, this latter was almost restored to its normal state, while the perception of temperature was altogether absent.—*Deutsche Klinik; Gazette Hebdomadaire; New York Med. Press.*

### NEW APPLICATION OF CHLOROFORM IN NEURALGIA AND IN CERTAIN RHEUMATIC COMPLAINTS.

During my residence at Singapore, East Indies, I was at one time in the habit of using liquor ammoniæ to produce an immediate blister, when instantaneous counter-irritation was thought necessary in certain cerebral affections, etc.—a piece of lint soaked in ammonia being applied to the part, and covered with oil-silk, when in a few minutes so much irritation was produced as to raise a blister. In administering chloroform to my patients, I noticed that their lips were often partially blistered by it; and recollecting the mode of using the ammonia, I thought of trying the chloroform in the same way, but found that neither oil-silk nor gutta percha tissue would answer. I then used a watch-glass to cover the lint soaked in it, and with the best effect.

The manner of application is to take a piece of lint, a little less in size than the watch-glass to be used (which need not be more than two inches in diameter), put it on the hollow side of the glass, pour on it a few drops of chloroform sufficient to saturate it, and then to apply it at once to the part affected, keeping the edges of the glass closely applied to the skin by covering it with the hand, for the purpose of keeping it in position, as well as of assisting the evaporation of the chloroform. This may be done from five to ten minutes, according to the amount of irritation wished for.

The patient during this time will complain of the gradual increase of a burning sensation (not so severe as that produced by a mustard sinapism), which reaches its height in five minutes, and then abates, but does not entirely disappear for more than ten minutes.

To ensure the full operation of the remedy, it is necessary that the watch-glass be rather concave, that it be closely applied to the skin, and that the hand applied over it be sensibly warm. The immediate effect of the application is to remove all local pain in neuralgia, and relieve that of rheumatism.

Its effects on the skin are first a reddening of the cutis, which in some cases is followed by desquamation of the cuticle; but this depends on the part to which it is applied, and also upon the susceptibility of the individual. In some cases, if the application is prolonged, a dark brown stain remains, even for a week or ten days, the same effect as sometimes follows the use of a mustard sinapism.

In Singapore I have used chloroform after this fashion in various neuralgias of the face, in inflammations of the eye and ear, in one case of angina pectoris, in several cases of neuralgia affecting the abdominal parietes, in lumbago, dysmenorrhœa, and in pain attending congestion of the ovary, etc.

Personally, I can testify to its great efficacy in two severe attacks of rheumatic inflammation of the eyes, in which the pain came on periodically about 3 A. M., with such severity that I thought the loss of sight itself would be preferable to its continuance. All other remedies, such as blisters, leeches, opium externally and internally, belladonna, etc., were of no avail in soothing the pain; water, almost boiling, applied by a sponge, giving only a little relief. I then thought of this use of chloroform, remembering how much it had benefited my patients in other similar affections. The first night, the application of it to the temple relieved the pain in ten minutes; on its return the next night, the application again relieved it; and four times only was it required to remove completely the local pain; allowing, in the meantime, constitutional remedies to produce their effect. Since my return to this country, I have recommended this remedy on several occasions to persons suffering from neuralgia of the face and head, and always with the same good effects as in India; and the other evening one of my domestics was quickly and effectually relieved by it of a painful spasmodic contraction of the platysma myoides muscle, which prevented her raising her head from the chest. The chloroform was applied as directed, with immediate benefit, and next morning she was quite well, though in previous attacks several days elapsed before relief was obtained. I have mentioned this method to several medical men of this city, who have found it of great benefit; and that it may be more extensively known, is my reason for now bringing it before the profession.

Dr. Keiller mentioned that this plan had been tried with success in his wards.

Dr. Wright had used chloroform for similar purposes, by pouring it into a bottle containing blotting-paper, and applying it over the affected painful part. He has found it sometimes produce vesication, and leave a mark on the skin; but it had been effectual in removing pain.

Mr. Little has received the following letter from Dr. Sclanders, House Physician to Dr. Keiller in the Royal Infirmary:

ROYAL INFIRMARY, March 14, 1860.

MY DEAR SIR,—I have much pleasure in giving you the result of my experience in regard to the external application of chloroform in the way proposed by you. Soon after you made me aware of it, I saw a friend of mine, who suffered frequently from neuralgia of the left forehead. I proposed the remedy to him, and with the effect of immediately removing the pain. Owing to my having kept it too long applied, vesication ensued. Since then he has had no return.

I have since used it in several cases of neuralgia of the ovary and pleurodynia, as also in two cases of rheumatic pains in the joints, with marked benefit.

I am, yours truly, ALEX. SCLANDERS.

DR. LITTLE. [*Edinburgh Medical Journal.*

---

The *Journal de Chimie Mèdicale* contains an account of the discovery of a new and powerful sedative in neuralgia, just discovered by Dr. Field. The substance used is nitrate of oxyde and glycile, and is obtained by treating glycerine at a low temperature with sulphuric or nitric acid. One drop mixed with ninety-nine drops of spirits of wine constitutes the first dilution. It has been tried upon animals and patients with remarkable effect. A case of neuralgia, in an old lady, which had resisted every known remedy, was completely cured by this new agent. It has also been tried in dental neuralgia with equal success.—*New York Daily Times.*

